# Single-Cell Sequencing of Immune Cell Heterogeneity in IgG4-Related Disease

**DOI:** 10.3389/fimmu.2022.904288

**Published:** 2022-05-27

**Authors:** Xunyao Wu, Yu Peng, Jieqiong Li, Panpan Zhang, Zheng Liu, Hui Lu, Linyi Peng, Jiaxin Zhou, Yunyun Fei, Xiaofeng Zeng, Yan Zhao, Wen Zhang

**Affiliations:** ^1^ Department of Rheumatology, Peking Union Medical College Hospital, Chinese Academy of Medical Science & Peking Union Medical College, National Clinical Research Center for Dermatologic and Immunologic Diseases, State Key Laboratory of Complex Severe and Rare Diseases, Beijing, China; ^2^ Clinical Biobank, Department of Medical Research Center, Peking Union Medical College Hospital, Chinese Academy of Medical Sciences and Peking Union Medical College, Beijing, China

**Keywords:** IgG4-RD, single-cell sequencing, immune cells, B cells, myeloid cells, T cells

## Abstract

**Background:**

The IgG4-related disease (IgG4-RD) is an immune-mediated disorder with fibrotic manifestations. However, the transcriptional profiles of immune cell subsets at single-cell level are unknown. Herein, single-cell sequencing was used to assess the specific cell subpopulations and pathways in peripheral blood mononuclear cells (PBMCs) of IgG4-RD.

**Methods:**

Single-cell sequencing was performed using the PBMCs from four patients with IgG4-RD and three healthy controls (HCs). Functional enrichment and cell analysis were performed through re-clustering of PBMCs to assess functional pathways and intercellular communication networks in IgG4-RD. Western blot and flow cytometry were used to verify sequencing and functional enrichment results.

**Results:**

Four major cell types and 21 subtypes were identified. Further subclustering demonstrated that plasma B-cell proportions increased with increasing glycolysis/gluconeogenesis activity in IgG4-RD. Re-clustering of myeloid cells showed that *EGR1* and *CD36* expressions were significantly increased in CD14^+^ monocytes of IgG4-RD, as validated by Western blot analysis. Moreover, tumor necrosis factor (TNF) production pathways were positively regulated in CD14^+^ monocytes of IgG4-RD. *In vitro* stimulation showed that CD14^+^ monocytes of IgG4-RD could secrete higher levels of TNF-α . Notably, the proportions of CD8 central memory T (TCM) and TIGIT^+^ CD8 cytotoxic T (CTL) increased in patients with IgG4-RD compared with HCs. Further interaction analysis showed that B cell activation factor (BAFF) signaling pathways were enriched from myeloid cells subsets to B cells.

**Conclusion:**

This study enhances the understanding of the cellular heterogeneity and transcriptional features involved in the pathogenesis of IgG4-RD, providing key clinical implications.

## Background

IgG4-related disease (IgG4-RD) is characterized by high IgG4 concentrations in serum or infiltration of IgG4^+^ plasma cells in affected tissues (1). IgG4-RD can affect any organ, causing immune-mediated fibrotic manifestations (1, 2). Currently, glucocorticoids are considered the first-line treatment for IgG4-RD (3). However, new therapeutic strategies are needed due to the potential toxicity and side effects of long-term glucocorticoid treatment.

The pathogenesis of IgG4-RD has been thoroughly assessed in recent years. The IgG4-RD is an immune-mediated disorder with diverse autoimmune features. Moreover, anti-galectin-3 autoantibodies have been identified in some patients with IgG4-RD (4). Lanzillotta et al. showed that peripheral plasmablast and plasma B cells are increased in patients with active untreated IgG4-RD. However, they showed that glucocorticoid treatment could alleviate the effect (5, 6). Rituximab can deplete B cells, thus substantially improving symptom, further validating the pathogenicity of B cells in IgG4-RD (7, 8). Self-reactive cytotoxic CD4 T cells, T follicular helper cells, and regulatory T cells can promote fibrosis and IgG4 production by B cells in patients with IgG4-RD (9–12). However, the role of dysregulated immune cells in the pathogenesis of IgG4-RD is unclear. Furthermore, the phenotype, function, and pathogenic heterogeneity of immune cells in IgG4-RD are unknown.

Single-cell sequencing (scRNA-seq) is a novel tool that can assess the heterogeneity of different immune subpopulations. Moreover, the transcriptome analysis at a single-cell or a cell-type level can be used to assess the expression of key genes and intracellular signaling pathways involved in the disease progression. Herein, scRNA-seq analysis of PBMCs from patients with IgG4-RD was used to assess specific cell subpopulations and pathways involved in IgG4 and chronic inflammation.

## Materials and Methods

### Recruitment and Ethics

This study included patients diagnosed with IgG4-RD based on the 2019 American College of Rheumatology/European League Against Rheumatism (ACR/EULAR) Classification Criteria for IgG4-RD (13) and healthy controls (HCs) at Peking Union Medical College Hospital (PUMCH). All the enrolled patients were treatment-naïve and they have not received any treatment before. This study was approved by the ethics committee of PUMCH (approval number: JS-3389), and informed consent was obtained from each patient. The detailed information on the procedure is provided in **Supplementary Table 1**.

### Single-Cell Suspension Preparation and Single-Cell Sequencing

PBMCs were obtained *via* Ficoll gradients (Human Lymphocyte Separation Medium, Dakewe, China), then centrifuged at 1,800 rpm and room temperature for 20 min. The cells were washed using 1× phosphate buffered saline (PBS) containing 0.5% fetal bovine serum (FBS) and then resuspended in 1× PBS containing 0.5% FBS. An Automated Cell Counter (Bio-Rad, TC20) was used to determine cell count and viability. Briefly, complementary DNA (cDNA) was synthesized from the cells and amplified using the v2 single-cell reagent kit (10X Genomics) following the manufacturer’s instructions. The sequencing library was constructed using simplified cDNA and then sequenced on Illumina (NovaSeq, Novogene).

### Raw Data Processing and Combination

The cellranger v2.1.0 pipeline was used to generate and align the raw gene expression matrix of each sample to the hg19 genome and transcriptome. The samples were combined using the Seurat package (v.3.0.0) based on the integration methods described at https://satijalab.org/seurat/v3.0/integration.html (14).

### Single-Cell RNA-Seq Data Processing

**Figure 1:**
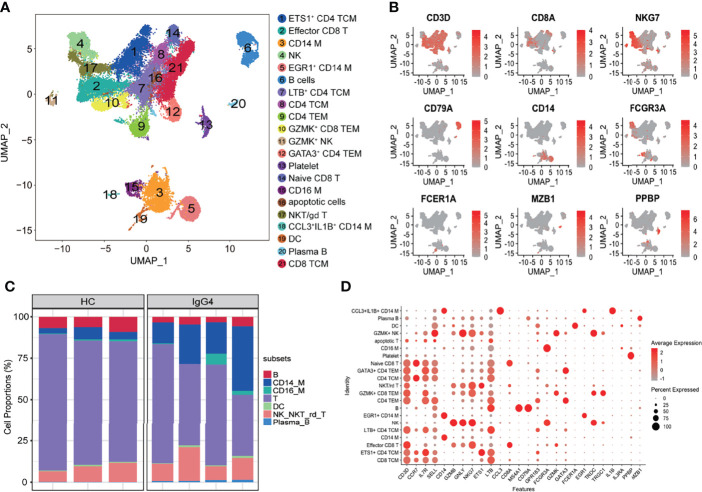
Overview of the clustering and annotation of the sc-RNA sequencing data for IgG4-RD. **(A)** UMAP representation of 47,219 single cells from HCs (n = 3) and IgG4-RD (n = 4), showing the formation of 21 clusters. **(B)** Canonical cell markers used to label major cell types represented in the UMAP plot. The legend is labeled in log scale. **(C)** Bar plot showing cluster abundance of major cell types across all samples. **(D)** Bubble heatmap showing the selected markers annotating specific cell types. The size of the dot indicates the fraction of expressing cells, colored according to z-score–normalized expression levels.

Single-cell RNA-seq data processing was conducted using R software (v.3.5.3) *via* the Seurat package (v.3.0.0). The following cells were filtered out: (1) cells with >10% transcripts mapping to the mitochondrial genes; (2) cells with fewer than 500 total unique transcripts; and (3) cells with a unique gene count of more than 3,500 genes. NormalizeData function was used to normalize the data. FindVariableFeatures function was used to calculate 2,000 features with high cell-to-cell variation. The RunPCA function was used to reduce the dimensionality of the datasets at default parameters on linear-transformation scaled data generated by the ScaleData function. FindNeighbors and FindClusters functions were used to perform nonlinear dimensional reduction *via* the RunUMAP function (dims = 1:30, resolution = 0.3). The details of the Seurat analyses performed in this work can be found in the website tutorial (https://satijalab.org/seurat/v3.0/pbmc3k_tutorial.html). Cell identity was annotated using the markers shown in **Figures 1B, C**. The uniform manifold approximation and projection (UMAP) were used for visualization.

### Subclustering of B Cells, Myeloid Cells, and T Cells

Subclustering analysis was conducted using B cells, myeloid cells (monocytes and dendritic cells), and T cells from PBMCs. The genes were scaled to unit variance after integration. Scaling, principal component analysis (PCA), and clustering were conducted as described above. Doublet clusters were removed under the following criteria: (1) B subclusters with the mean expression of CD3D, CD14, or PPBP > 0.1; (2) myeloid subclusters with the mean expression of CD3D, CD79A, or PPBP > 0.1; and (3) T subclusters with the mean expression of CD79A, CD14, or PPBP > 0.1.

### Identification of Differentially Expressed Genes

The differentially expressed genes (DEGs) were identified *via* FindMarkers function in Seurat using the parameters “test.use = wilcox” by default. The false discovery rate (FDR) was estimated using the Benjamini–Hochberg method. DEGs were filtered using a minimum log_2_ (fold change) of 0 and a maximum adjusted p-value of 0.05 and then ranked based on the average log2 (fold change) and FDR.

### Gene Function Enrichment Analysis

Gene Set Enrichment Analysis (GSEA) (version 3.0) was used to analyze the enriched signaling pathways of plasma B and Mem-unsw B-cell subtypes. Signaling pathways with a threshold of p-value < 0.05 were considered significantly enriched (15). For myeloid or T/Natural killer (NK) cells cells, enrichment analysis for the functions of the DEGs was conducted using the clusterProfiler (v3.12.0) package. Moreover, gene sets were based on Gene Ontology (GO) terms (16).

### Cell and Cell Interaction

CellChat was used to comprehensively assess the global communications among cells and quantitatively analyze intercellular communication networks (17). Briefly, the official workflow was followed, and the normalized data were loaded into *CellChat*. *CellChat* objects were created, and *CellchatDB.human* was used to set the secreted signaling pathways as the database.

### Purification of Monocytes and *In Vitro* Stimulation

Peripheral blood from patients and HCs were collected in ethylene diamine tetraacetic acid tubes. The blood was then diluted with 1× PBS at a ratio of 1:1, put on the Ficoll density gradient (Dakewe, China) and then centrifuged at 1,800 rpm and 24°C for 20 min. The peripheral blood mononuclear cells (PBMCs) were collected at the interface layer, washed with PBS (300*g* for 10 min, 4°C), and then counted using cellmeter Auto T4 (Nexcelom Bioscience, USA). CD14 microbeads (Miltenyi Biotec, USA) were used to purify CD14^+^ monocytes following the manufacturer’s instructions. The purified CD14^+^ monocytes were counted through cellmeter Auto T4 (Nexcelom Bioscience, USA) and were resuspended in dulbecco’s modified eagle medium (DMEM) (Gibco, USA) containing 10% FBS (Gibco, USA) then seeded into 24-well plates (5 × 10^5^ per well) with recombinant interferon-γ (IFN-γ) (PeproTech, USA) at 37°C for 6 h. The supernatants were then collected for further analysis.

### TNF-α Detection

A human tumor necrosis factor–α (TNF-α) pre-coated ELISA kit (Dakewe, China) was used to detect TNF-α in supernatants following the manufacturer’s instructions. Briefly, diluted cytokine standards (100 µl), undiluted samples (100 µl), and biotinylated antibody (50 µl) were added to 96-well plates and incubated at room temperature for 3 h. The plates were washed thrice using wash buffer, and then, streptavidin–horseradish peroxidase (HRP) working solution was added to each well and incubated at room temperature for 20 min. The plates were washed again thrice, then 100 µl of Tetramethylbenzidine (TMB) was added to each well and incubated at room temperature for 20 min away from light. A stopping solution was also added. Thermo Scientific Multiskan FC (Thermo Fisher Scientific, USA) was used to measure absorbance at 450 nm.

## Western Blotting

Purified CD14^+^ monocytes were lysed with RIPA buffer (High, Solarbio Life Sciences, China) on ice for 30 min and then centrifuged at 12,000*g* for 10 min. A BCA protein assay kit (Thermo Scientific, USA) was used to detect protein concentration. Protein samples [5 µg per lane for CD36 and 10 µg per lane for early growth response-1 (EGR1)] were separated *via* sodium dodecylsulphate (SDS)–polyacrylamide gel electrophoresis and electrophoretically transferred to Immobilon™-P polyvinylidene difluoride membranes (MilliporeSigma, Germany). The membranes were blocked with QuickBlock™ Blocking Buffer (Beyotime, China) at room temperature for 1 h and then incubated with primary antibodies [EGR1 Rabbit mAb (Cell Signaling Technology, USA, 1:1,000), CD36 Rabbit mAb (Cell Signaling Technology, USA, 1:1,000), and β-Actin Mouse mAb (EASYBIO, China, 1:5,000)] at 4°C overnight. The membranes were washed and incubated with secondary antibody [HRP-conjugated (EASYBIO, China, 1:5,000)]. Chemiluminescent HRP Substrate (Millipore Sigma, Germany) were detected using immunoreactive bands. Images were obtained through Tanon 3000M. The relative expression of EGR1 and CD36 were analyzed using ImageJ software (US National Institutes of Health, Bethesda, MA, USA).

### Data Availability

The raw sequence data were deposited in the Genome Sequence Archive of the Beijing Institute of Genomics (BIG) Data Center, BIG, Chinese Academy of Sciences (accession code, HRA001555) and are publicly accessible at http://bigd.big.ac.cn/gsa-human. Other supporting raw data are available from the corresponding author upon reasonable request.

## Results

### An Overview of PBMC Composition in Patients with IgG4-RD

The scRNA-seq was used to analyze peripheral blood samples of three healthy individuals and four patients with IgG4-RD (**Supplementary Table 1**). A total of 47,219 cells were retained for subsequent analysis after filtering doublets and poor-quality cells (dead or dying cells). Un-supervised clustering followed by a two-dimensional UMAP identified 21 distinct subsets (**Figure 1A**). Four major populations, including T cells, NK cells, B cells, myeloid cells, were identified on the basis of canonical markers (*CD3D*, *CD8A*, *NKG7*, *CD79A*, *CD14*, *FCGR3A*, *FCER1A*, *MZB1*, and *PPBP*) (**Figure 1B**). Overall, patients with IgG4-RD had decreased B cells and increased CD14 monocytes and plasma B proportions (**Figure 1C**). Additional cluster-defining genes of each cluster are shown in **Figure 1D**.

### Transcriptional and Pathway Analysis of B-Cell Subsets in IgG4-RD

**Figure 2:**
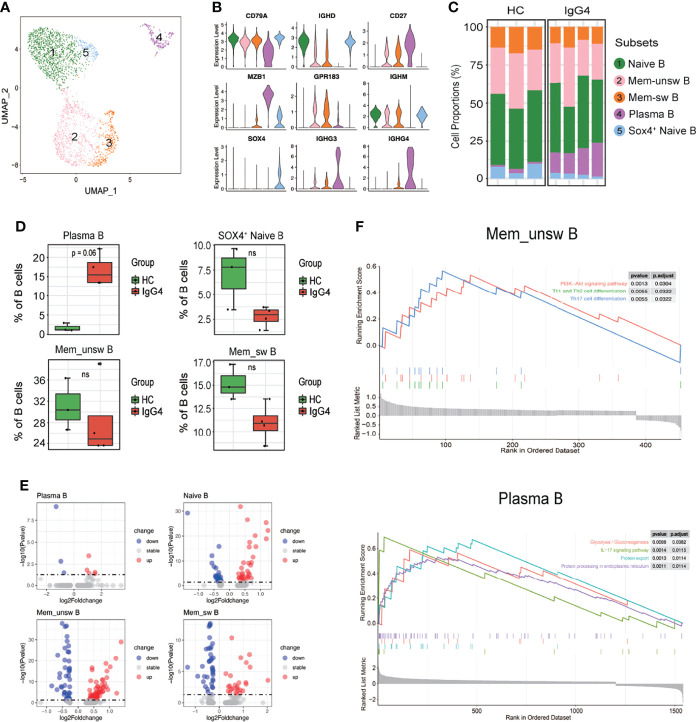
The heterogeneity and transcriptional features of B-cell subsets in IgG4-RD. **(A)** UMAP representation of 2,449 B cells, showing the formation of five clusters. **(B)** Violin plots showing expression distribution of canonical cell markers. **(C)** Bar plot showing cluster abundance of each B-cell type across all samples. **(D)** Percentages of four types of B-cell between HCs and patients with IgG4-RD. The *y*-axis shows the average percentage of B-cell subtypes in total B cells. Error bars are shown in mean ± SEM. Student *t*-test was used for the analysis, and P < 0.05 is considered as a significant difference, ns stands for nonsignificant. **(E)** Volcano plot showing the DEGs of B-cell subtypes between HCs and patients with IgG4-RD. **(F)** GSEA results showing the significantly enriched pathways in Memory-unswitched (Mem-unsw) B cells and Plasma B cells of patients with IgG4-RD.

Subclustering of three major populations (B cells, myeloid cells, and T/NK cells) was further conducted. IgG4-RD is characterized by increased IgG4-secreting B cells. This is the first study to report B-cell subpopulations in patients with IgG4-RD. B cells were subclustered into five major populations based on *CD79A*, *IGHD*, *CD27*, *MZB1*, *GPR183*, *IGHM*, *SOX4*, *IGHG3*, and *IGHM*: naïve B (cluster 1, *IGHD* ^+^ *CD27* ^−^), Mem-unsw B (cluster 2, memory-unswitched B cells, *IGHD* ^+^ *CD27* ^+^ *GPR183* ^+^), Mem-sw B (cluster 3, memory-switched B cells, *IGHD* ^+^ *CD27* ^+^ *GPR183* ^+^), plasma B (cluster 4, *MZB1* ^+^ *IGHG3* ^+^ *IGHG4* ^+^), and SOX4^+^ naïve B (cluster 5, *IGHD* ^+^ *CD27* ^−^ *SOX4* ^+^) (**Figures 2A, B**). Patients with IgG4-RD had increased plasma B levels (**Figures 2C, D**). The molecular differences of various B-cell subsets between HCs and patients with IgG4-RD were assessed using volcano plots (**Figure 2E**, **Supplementary Figure 1A**). There was only one differential gene, *IGHG3*, in SOX4^+^ naïve B-cell subsets between HCs and patients with IgG4-RD (**Supplementary Figures 1A, B**). Functional difference was assessed using GSEA. The top enriched GSEA items for the Mem_unsw B cells included “PI3K-Akt signaling pathway”, “Th1 and Th2 cell differentiation,” and “Th17 cell differentiation”, indicating the enhanced proliferation and ability to activate T-cell responses in patients with IgG4-RD (**Figure 2F**). The top enriched GSEA items for plasma B cells in patients with IgG4-RD included “glycolysis/gluconeogenesis”, “IL-17 signaling pathway”, “protein export”, and “protein processing in endoplasmic reticulum”, indicating that abnormal metabolism can promote antibody processing and secretion (**Figure 2F**).

### Pro-Inflammatory Gene Patterns of Myeloid Cells in IgG4-RD

**Figure 3:**
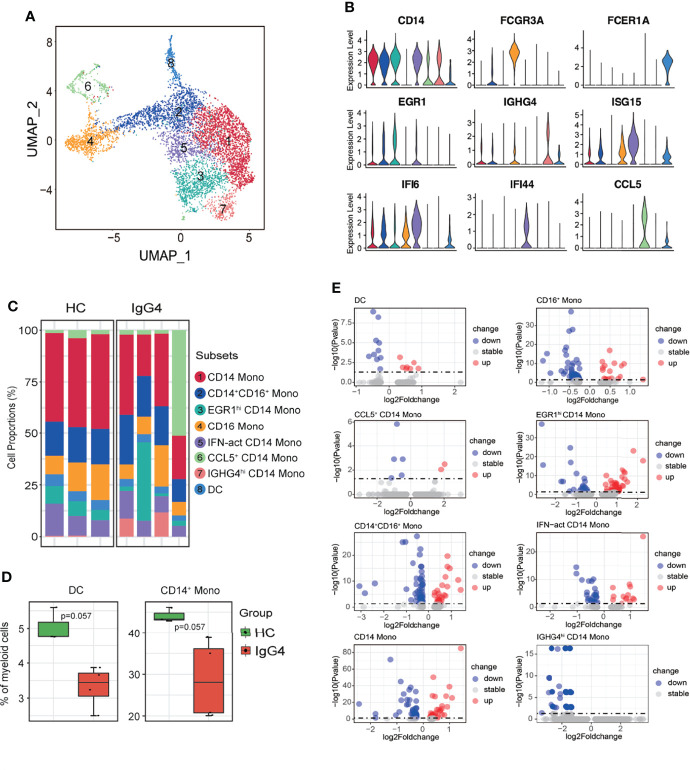
The heterogeneity and transcriptional features of myeloid cells in IgG4-RD. **(A)** UMAP representation of 6324 myeloid cells, showing the formation of 8 clusters. **(B)** Violin plots showing expression distribution of canonical cell markers. **(C)** Bar plot showing cluster abundance of each myeloid cell type across all samples. **(D)** Percentages of DC and CD14^+^ Mono between HCs and IgG4-RD. The *y*-axis shows the average percentage. Error bars are shown in mean ± SEM. Student *t*-test was used for the analysis, and *P* < 0.05 is considered a significant difference. **(E)** Volcano plot showing the DEGs of myeloid cell subtypes between HCs and patients with IgG4-RD. Data are expressed as mean ± SD.

**Figure 4:**
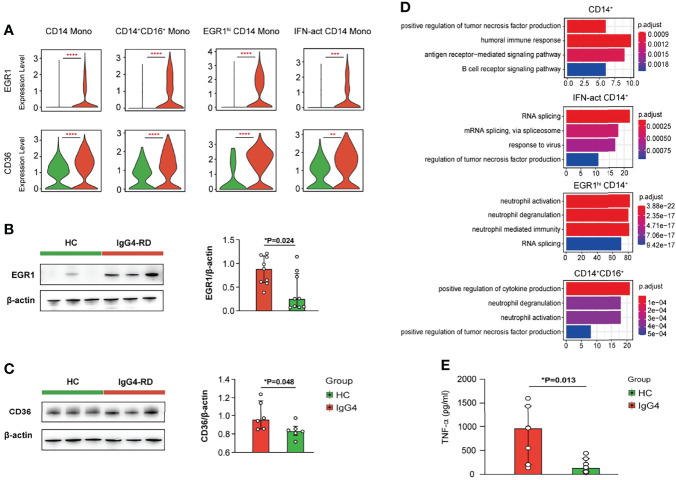
Functional analysis of myeloid cells in IgG4-RD. **(A)** Violin plots showing expression of *EGR1* and *CD36* in different CD14^+^ mono subtypes. **(B, C)** The comparison of EGR1 and CD36 expressions in purified CD14^+^ monocytes from HCs and patients with IgG4-RD. **(D)** GO analysis showing the biological process enriched in different CD14^+^ monocytes of patients with IgG4-RD. **(E)** CD14^+^ monocytes purified from PBMCs of HCs and patients with IgG4-RD and cultured for 12 h *in vitro*. The TNF-α in the supernatant was measured using ELISA. Data are expressed as mean ± SD, *P < 0.05, **P < 0.01, ***P < 0.001, and ****P < 0.0001.

Subclustering showed that myeloid cells are monocytes (mono) and dendritic cells (DCs). The monocytes were re-classified into seven subsets based on *CD14*, *FCGR3A*, *EGR1*, *IGHG4*, *ISG15*, *IFI6*, *IFI44*, and *CCL15*: CD14 Mono (*CD14*), CD14^+^CD16^+^ Mono (*CD14* and *FCGR3A*), EGR1^hi^ CD14 Mono (*EGR1* and *CD14*), CD16 Mono (*FCGR3A*), IFN-act CD14 Mono (*CD14*, *ISG15*, *IFI6*, and *IFI44*), CCL5^+^ CD14 Mono (*CCL5* and *CD14*), and IGHG4^hi^ CD14 Mono (*IGHG4* and *CD14*) (**Figures 3A–C**). The DC and CD14 Mono proportions were lower in myeloid cells of patients with IgG4-RD (**Figures 3C, D**). Similarly, the molecular differences of various myeloid subsets between HCs and patients with IgG4-RD were assessed using volcano plots (**Figure 3E**). The expressions of *EGR1* and *CD36* were significantly higher in CD14 Mono, CD14^+^CD16^+^ Mono, EGR1^hi^ CD14 Mono, and IFN-act CD14 Mono than in other subsets (**Figure 4A**). Western blot of purified CD14^+^ monocytes from HCs and patients with IgG4-RD showed that EGR1 and CD36 expressions were significantly increased in proteins of IgG4-RD–derived CD14^+^ classical monocytes (**Figures 4B, C**).

GO pathway analysis of DEGs between different myeloid subsets showed that TNF was uniformly and significantly upregulated in CD14^+^ Mono, IFN-act CD14^+^ Mono, and CD16^+^ Mono of patients with IgG4-RD (**Figure 4D**). *In vitro* stimulation analysis indicated that CD14^+^ monocytes of IgG4-RD secreted higher levels of TNF-α (**Figure 4E**). In addition, DEGs in EGR1^hi^ CD14 Mono and CD14^+^CD16^+^ Mono from patients with IgG4-RD were positively enriched in neutrophil activation and neutrophil degranulation-related pathways (**Figure 4D**).

### Transcriptional Features of NK/T-Cell Subsets in Patients With IgG4-RD

Subclustering of NK/T cells obtained 12 subsets based on the canonical NK/T-cell markers: two subtypes of NK cells, six subtypes of CD4^+^ T cells, and four subtypes of CD8^+^ T cells (**Supplementary Figure 2**). Further subclustering of NK cells identified four NK/NKT-cell clusters: PTGDS^+^ NK (*PTGDS* and *NKG7*), NKT (*NKG7* and *CD3D*), GZMK^+^IGFBP4^+^ NK (*GZMK*, *IGFBP4*, and *NKG7*), and TIGIT^+^ NKT (*TIGIT*, *NKG7*, and *CD3D*) (**Supplementary Figures 3A, B**). The proportions of each NK/NKT subsets were similar between HCs and patients with IgG4-RD (**Supplementary Figure 3C**). Moreover, GO analysis indicated that natural killer cell-mediated cytotoxicity and immunity were decreased in NK or NKT cells of patients with IgG4-RD (**Supplementary Figure 3D**).

**Figure 5:**
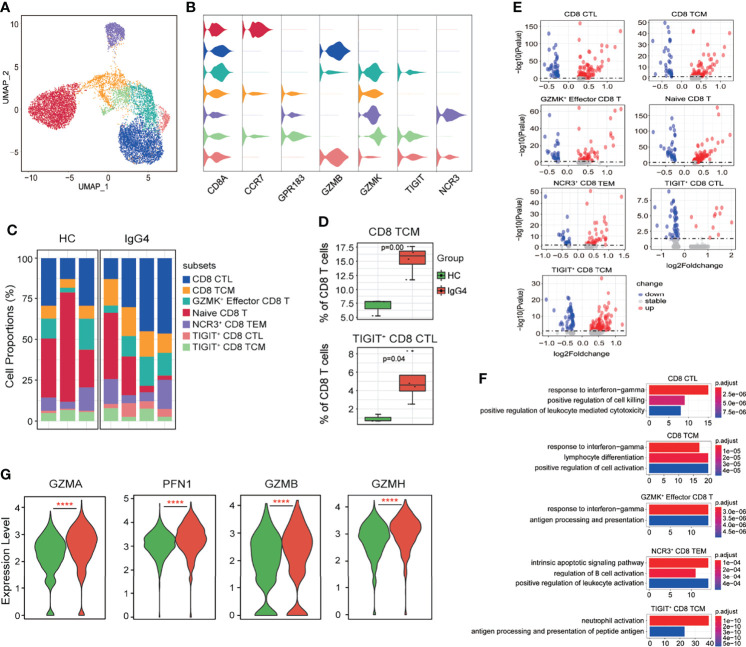
The heterogeneity and transcriptional features of CD8 T-cell subsets in IgG4-RD. **(A)** UMAP representation of 11,864 CD8 T cells, showing the formation of seven clusters. **(B)** Violin plots showing expression distribution of canonical cell markers. **(C)** Bar plot showing cluster abundance of each CD8 T-cell type across all samples. **(D)** Percentages of two types of CD8 T-cell between HCs and IgG4-RD. The *y*-axis shows the average percentage of CD8 T-cell subtypes in total CD8 T cells. Error bars are shown in mean ± SEM. Student *t*-test was used for the analysis, and *P* < 0.05 is considered as a significant difference. **(E)** Volcano plot showing the DEGs of CD8 T-cell subtypes between HCs and patients with IgG4-RD. **(F)** Violin plots showing expression of *GZMA*, *PFN1*, *GZMB*, and *GZMH* in CD8 CTL. **(G)** GO analysis showing the biological process enriched in different CD8 T-cell subtypes of patients with IgG4-RD, ****P < 0.0001.

Subclustering of CD8^+^ T cells obtained seven subtypes: CD8 CTL (cytotoxic lymphocytes, *CD8A*, and *GZMB)*, CD8 TCM (central memory T, *CD8A*, *CCR7*, and *GRP183*), *GZMK* ^+^ Effector CD8 T (*CD8A* and *GZMK*), naïve CD8 T (*CD8A* and *CCR7)*, *NCR3* ^+^ CD8 effector memory T (TEM, *NCR3*, *CD8A*, and *GPR183*), *TIGIT* ^+^ CD8 CTL (*TIGIT*, *CD8A*, and *GZMB*), and *TIGIT* ^+^ CD8 TCM (*TIGIT*, *CD8A*, *CCR7*, and *GPR183*) (**Figures 5A–C**). Notably, CD8 TCM and TIGIT^+^ CD8 CTL proportions were increased in patients with IgG4-RD compared with HCs (**Figures 5C, D**). The DEGs of various CD8^+^ T subsets were assessed, and then, GO analysis was conducted based on the DEGs (**Figure 5E**). The CD8 CTL, CD8 TCM, and *GZMK* ^+^ effector CD8 T-cell subsets of patients with IgG4-RD were associated with significantly enhanced responses to IFN-γ, antigen processing and presentation, and lymphocyte differentiation (**Figure 5F**). Cell killing and leukocyte-mediated cytotoxicity were significantly positively regulated in CD8 CTL of patients with IgG4-RD (**Figure 5F**). Similarly, the levels of cytotoxicity-related markers, including *GZMA*, *PFN1*, *GZMB*, and *GZMH*, were significantly higher in CD8 CTL of patients with IgG4-RD than in HCs (**Figure 5G**). Moreover, B-cell and neutrophil activation was enhanced in *NCR3* ^+^ CD8 TEM and *TIGIT* ^+^ CD8 TCM of patients with IgG4-RD, respectively (**Figure 5F**).

**Figure 6:**
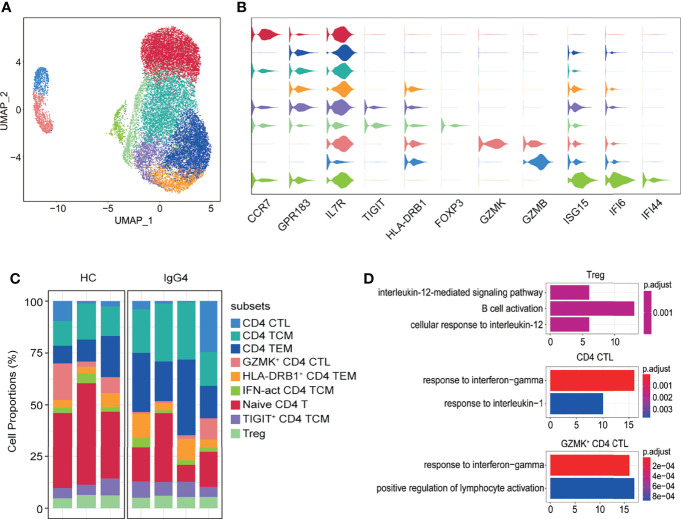
The heterogeneity and transcriptional features of CD4 T-cell subsets in IgG4-RD. **(A)** UMAP representation of 19,629 CD4 T cells, showing the formation of nine clusters. **(B)** Violin plots showing expression distribution of canonical cell markers. **(C)** Bar plot showing cluster abundance of each CD4 T-cell type across all samples. **(D)** GO analysis showing the biological process enriched in Treg, CD4 CTL, and *GZMK* ^+^ CD4 CTL of patients with IgG4-RD.

CD4^+^ T cells were further subclustered into nine subtypes: CD4 CTL (*GZMB*), CD4 TCM (*CCR7* and *GPR183*), CD4 TEM (*IL7R* and *GPR183*), GZMK^+^ CD4 CTL (*GZMK* and *GZMB*), *HLA-DRB1* ^+^ CD4 TEM (*HLA-DRB1*, *IL7R*, and *GPR183)*, IFN-act CD4 TCM (*ISG15*, *IFI6*, *IFI44*, *CCR7*, and *GPR183*), naïve CD4 T (*CCR7*), *TIGIT* ^+^ CD4 TCM (*TIGIT*, *CCR7*, and *GPR183*), and Treg (*FOXP3*) (**Figure 6A–C**). The abundance of CD4^+^ T subtypes was similar between HCs and patients with IgG4-RD (**Figure 6C**). The functional differences between various CD4 T subtypes were evaluated *via* GO analysis. Cellular response to IL-12 and B-cell activation were increased in Tregs of patients with IgG4-RD (**Figure 6D**). Moreover, CD4 CTL and *GZMK* ^+^ CD4 CTL from patients with IgG4-RD were more likely to respond to IFN-γ and IL-1 (**Figure 6D**).

### Global Analysis of Immune Cell Communications in Patients With IgG4-RD

The cellular interactions between immune cells play critical roles in cell activation, eventually leading to disease symptoms in patients with IgG4-RD. Herein, *CellChat* was used to investigate the putative interactions between immune cells in patients with IgG4-RD.

**Figure 7:**
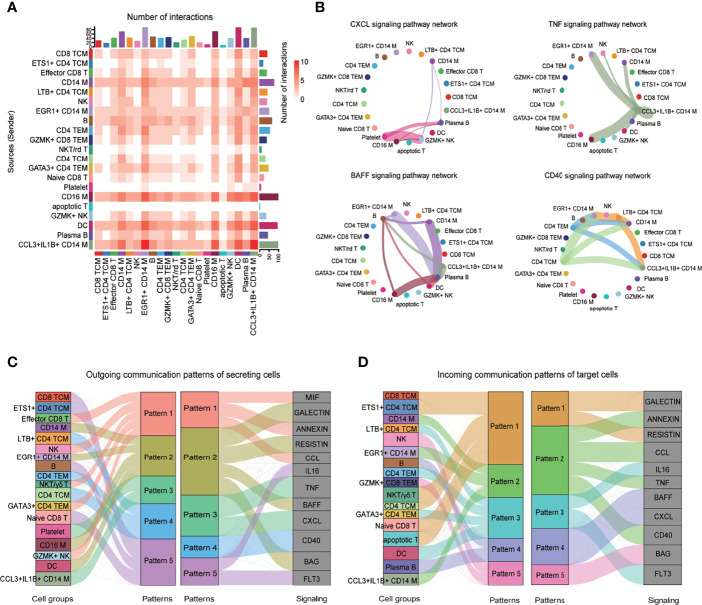
CellChat analysis of the communications among immune cells. **(A)** Heatmap showing the number of interactions of different cell subsets in PBMCs. **(B)** Circle plot showing the inferred intercellular communication network for CXCL, TNF, BAFF, and CD40 signaling among different cell subsets. The number of cells in each cluster is proportional to the circle size, and the line thickness represents the strength of signaling. **(C)** The inferred outgoing communication patterns of secreting cells, which shows the correspondence between the inferred latent patterns and cell groups, and signaling pathways. The thickness of the flow indicates the contribution of the cell group or signaling pathway to each latent pattern. **(D)** The inferred incoming communication patterns of target cells.

Interaction events were used to calculate the interaction times for each immune cell type. Interaction between myeloid cells and B cells was higher than in T cells subset (**Figure 7A**). *CellChat* was also used to identify significant pathways among the immune cells group. However, this study focused on the CXCL, TNF, B-cell–activating factor (BAFF), and CD40 pathways (**Figure 7B**). BAFF signaling pathways were enriched in myeloid cell subsets to B cells (**Figure 7B**). CD40 signaling pathway was the most enriched from CD4^+^ T cells to CD14^+^ monocytes (**Figure 7B**). Notably, *CellChat* also predicted that myeloid cells were key sources and mediators for CXCL and TNF signaling pathways (**Figure 7B**). *CellChat* was also used to analyze the communication patterns in different cell groups. Several outgoing effector T/NK cells were characterized by pattern #1, representing multiple pathways, including MIF, ANNEXIN, and CCL signaling pathways (**Figure 7C**). The outgoing of myeloid cells was characterized by pattern #2, representing mainly GALECTIN, RESISTIN, BAFF, and BAG signaling pathways (**Figure 7C**). Moreover, the communication patterns of target cells showed that the incoming effector T/NK cells signaling was dominated by pattern #1, #3, and #5, mainly representing GALECTIN, RESISTIN, IL16, FLT3, and BAG signaling pathways (**Figure 7D**). Most incoming CD14 Mono signaling were dominated by pattern #2, representing ANNEXIN, CCL, TNF, and CD40 signaling pathways (**Figure 7D**). Notably, the incoming B and plasma B signaling were characterized by pattern #4, representing BAFF and CXCL signaling pathways (**Figure 7D**). Together, the interaction analysis highlights the role of myeloid cells in promoting B-cell over-activation through BAFF signaling pathway in patients with IgG4-RD.

## Discussion

IgG4-RD is an immune-mediated fibrotic disease. Previous studies have reported the characteristics of immune responses in IgG4-RD disease, enhancing the understanding of potential immune pathogenesis of IgG4-RD disease. However, the cellular and molecular immune responses in IgG4-RD are unclear. Herein, the immunological landscape profiles in patients with IgG4-RD at single-cell resolution were assessed to reveal the critical factors and pathways involved in immune pathogenesis.

We observed reduced B-cell proportions at the single cell level. Our observation was in consistent with our recent published study in which we observed decreased percentage of CD19+ B cells in patients with IgG4-RD by flow cytometry (18). In addition, a previous study by Lanzillotta et al. (19) addressed decreased B cells absolute counts in patients with IgG4-RD compared with HCs. Plasma B-cell levels were increased in patients with IgG4-RD, consistent with previous findings (5, 20). Interestingly, “glycolysis/gluconeogenesis”, “protein export”, and “protein processing in endoplasmic reticulum” pathways were enriched in plasma B cells from patients with IgG4-RD. A previous study showed that glycolysis activity is correlated with plasmablast differentiation and disease activity (21). However, another study performed by Alvise Berti et al. (22) performed positron emission tomography (PET)/computerized tomography (CT) to measure [18F] Fluorodeoxyglucose (18F-FDG) uptake and found that circulating plasmablasts were inversely correlated with the total lesion glycolysis. Therefore, whether increased glycolysis or gluconeogenesis activity can induce class-switching and differentiation of plasma B cells, promoting antibody processing and production in patients with IgG4-RD needs further investigation.

Notably, the analysis of the incoming signaling *via CellChat* predicted that BAFF signaling pathway, mediated by myeloid cells, was dominant in B and plasma B subsets. BAFF is a key B-cell survival factor. BAFF overexpression is associated with autoantibody-related autoimmune diseases, such as systemic lupus erythematosus (SLE), primary sjogren’s syndrome (pSS) patients, IgA nephropathy, and rheumatoid arthritis (23–25). Clinical trials have used therapeutic monoclonal antibody neutralizing BAFF, belimumab, in recent years. Belimumab can be used as a targeted therapy for SLE (26–28). A previous study showed that the serum levels of BAFF and APRIL are significantly higher in IgG4-RD and pSS than in HCs (29). A recent study also demonstrated that BAFF produced by neutrophils and dendritic cells enhances antibody responses (30). Herein, myeloid cells from BAFF promoted class-switching and differentiation of B cells to IgG4-producing plasma B cells in patients with IgG4-RD. Similarly, a previous study found that IgG4-RD–derived monocytes can induce IgG4 production of HC-derived B cells in a BAFF-dependent and T cell-independent manner (31). Therefore, monoclonal antibody neutralizing BAFF can be used for the clinical treatment of IgG4-RD.

Macrophages play a role in IgG4-RD initiation. Previous studies have shown that CD163^+^ M2 macrophages are activated by TLR7, accumulated in multiple organs of patients with IgG4-RD, thus promoting fibrotic phenotype by producing CCL18 and IL-10 or activating T helper type 2 (Th2) immune response *via* IL-33 (32–34). Herein, increased TNF production in CD14^+^ monocytes promoted activation of NK cells and also acted as an autocrine to activate themselves. Moreover, EGR1 and CD36 expressions were significantly higher in CD14^+^ monocytes from IgG4-RD than those from HCs. A previous study showed that Egr-1 is significantly upregulated in the skin lesions of psoriasis patients and promotes TNF-α production (35). CD36 is a scavenger receptor. Macrophage CD36 can interact with oxidized low-density lipoprotein (oxLDL), trigger signaling cascades for inflammatory response, and is involved in atherosclerosis (36, 37). However, future studies should assess the mechanisms underlying the role of EGR1 and CD36 in promoting TNF production in patients with IgG4-RD.

Moreover, Th2 cells and Tregs play crucial roles in IgG4-RD. The number of Th2 cells is correlated with elevated serum IgG4 levels, IL-4, plasmablast counts, and disease activity (38, 39). PD-1^+^CXCR5^-^ circulating Tfh cell populations are significantly increased in patients with IgG4-RD than those in healthy volunteers and are correlated with IgG4 class switching and clinical manifestations of IgG4-RD (40–43). A previous study also found that the frequency of circulating Tfh is increased in the peripheral blood and involved tissue of IgG4-RD. An *in vitro* co-culture study showed that cTfh cells from IgG4-RD can facilitate B-cell proliferation and enhance the differentiation of naïve B cells into switched memory B cells and plasmablast/plasma cells (44). Expanded cytotoxic CD4^+^ T cells have been detected in patients with IgG4-RD. Moreover, SLAM7^+^granzyme A^+^IL-1β^+^TGF-β1^+^CD4 CTLs secreting IFN-γ are the dominant T cells infiltrating inflamed IgG4-RD tissue site (45, 46). Herein, response to IFN-γ was enhanced in both effector CD4 and CD8 T subsets. Moreover, some T-cell subsets in IgG4-RD activated B cells and neutrophils. Therefore, the role of IFN-γ and the interaction of T cells with neutrophils in patients with IgG4-RD should be assessed.

However, this study had a limited sample size, which may result in low resolution of further subclustering. Therefore, further studies with large samples and fibrotic tissues are needed to explore how immune cells promote fibrotic lesions of patients with IgG4-RD. Moreover, not all mechanistic studies were performed to validate the findings at the single cell level in our present study. Therefore, further efforts are still needed to validate the phenomenon that we observed at present study, e.g., whether enhanced glycolysis ability promote abnormal B-cell class-switching and antibody production, the role of increased EGR1 expression in monocytes, whether the involved pathways are actually enriched and their role in T/NK cells functions of patients with IgG4-RD.

In summary, this study enhances the understanding of the role of immune cells, thus providing new potential therapeutic targets for the treatment of patients with IgG4-RD.

## Data Availability Statement

The datasets presented in this study can be found in online repositories. The name of the repository and accession number can be found below: Genome Sequence Archive of the Beijing Institute of Genomics (BIG) Data Center, BIG, Chinese Academy of Sciences (http://bigd.big.ac.cn/gsa-human); accession code HRA001555.

## Ethics Statement

The studies involving human participants were reviewed and approved by the ethics committee of Peking Union Medical College Hospital (approval number: JS-3389). The patients/participants provided their written informed consent to participate in this study.

## Author Contributions

XW performed study design, literature search, data analysis, data interpretation, statistical analyses, and manuscript writing. YP performed the experiments, analyzed patients’ clinical data, and helped write the manuscript. JL recruited the patients, performed statistical analyses, and helped write the manuscript. PZ, ZL, and HL helped recruit the patients and conducted statistical analyses. LP, JZ, YF, and XZ helped recruit the patients. YZ and WZ conceived the study, provided patient samples, supervised experiments, and revised the manuscript. All authors contributed to the article and approved the submitted version.

## Funding

This work was supported by the National Natural Science Foundation of China (81771757, 82071839, and 81971544), Capital’s Funds for Health Improvement and Research (No. 2020-2-4017), CAMS Innovation Fund for Medical Sciences (CIFMS 2021-1-I2M-003), and Beijing Municipal Science and Technology Commission (No. Z201100005520023).

## Conflict of Interest

The authors declare that the research was conducted in the absence of any commercial or financial relationships that could be construed as a potential conflict of interest.

## Publisher’s Note

All claims expressed in this article are solely those of the authors and do not necessarily represent those of their affiliated organizations, or those of the publisher, the editors and the reviewers. Any product that may be evaluated in this article, or claim that may be made by its manufacturer, is not guaranteed or endorsed by the publisher.
