# Supplementary material for: Single-Cell Sequencing of Immune Cell Heterogeneity in IgG4-Related Disease
Source: Front Immunol. 2022 May 27;13:904288. doi: 10.3389/fimmu.2022.904288 (PMC9184520; doi:10.3389/fimmu.2022.904288)
Supplement: Supplementary file 1 [file DataSheet_1.docx]

**Table S1. The detailed clinical features of the enrolled IgG4-RD patients (n=20) in this study.**

|  | **Values** | **Normal ranges** |
| --- | --- | --- |
| **Demographic features** | | |
| Sex (Male : Female) | 1.9 : 1 | N. A |
| Age at onset (Mean ± S.D.) | 57.55±14.61 | N. A |
| Disease duration (Month; Median, IQR) | 24 (6-81) | N. A |
| Affected organ numbers (Mean ± S.D.) | 3.4±1.7 | N. A |
| RI scores (Mean ± S.D.) | 7.3±3.39 | N. A |
| Treatment naïve (n%) | 20/20, 100% | N. A |
| **Serological features (Median, IQR)** | | |
| IgG4 (mg/L) | 9160 (3987.5-20275) | 80-1400 |
| IgG (g/L) | 21.17 (15.82-24.81) | 7.00-17.00 |
| IgA (g/L) | 1.80 (1.39-3.18) | 0.70-4.00 |
| IgM (g/L) | 0.81 (0.6-1.14) | 0.40-2.00 |
| IgE (KU/L) | 340.5 (214.5-524.25) | < 60 |
| Eosinophils (10^9^/L) | 0.21 (0.11-0.37) | 0.02-0.5 |
| ESR (mm/h) | 19 (9-30) | 0-20 |
| CRP (mg/L) | 1.04 (0.38-2.70) | < 8.0 |
| C3 (g/L) | 0.78 (0.64-1.08) | 0.73-1.46 |
| C4 (g/L) | 0.12 (0.07-0.19) | 0.1-0.4 |
| ANCA autoantibodies (Negative%) | 20/20, 100% | N. A |
| **Organ involvements (n%)** | |  |
| Lacrimal gland | 11 (55%) | N. A |
| Parotid gland | 3 (15%) | N. A |
| Submandibular gland | 14 (70%) | N. A |
| Pancreas | 6 (30%) | N. A |
| Bile duct | 2 (10%) | N. A |
| Lung | 6 (30%) | N. A |
| Kidney | 2 (10%) | N. A |
| Retroperitoneum | 3 (15%) | N. A |
| Paranasal sinus | 5 (25%) | N. A |
| Lymph node | 11 (55%) | N. A |

**S.D. represented for Standard deviation, IQR represented for interquartile range.**

**RI scores was the IgG4-RD responder index scores; IgG, IgA, IgM, IgG4, IgE were immunoglobulin G, A, M, G4 and E. ESR was Erythrocyte Sedimentation Rate, CRP was C-reactive protein, RF was rheumatoid factor and C3/4 represented for complement 3/4.**

**Table S2. The detailed clinical information of the four enrolled IgG4-RD patients used for scRNA-seq.**

|  | **Patient 1** | **Patient 2** | **Patient 3** | **Patient 4** |
| --- | --- | --- | --- | --- |
| **Demographic features** | | |  |  |
| Sex | Male | Male | Male | Female |
| Age at onset | 60 | 61 | 55 | 21 |
| Disease duration (Month) | 14 | 84 | 96 | 48 |
| Affected organ numbers | 4 | 4 | 3 | 3 |
| RI scores | 8 | 8 | 6 | 6 |
| Treatment naïve | Yes | Yes | Yes | Yes |
| **Serological features** | | |  |  |
| IgG4 (mg/L) | 5630 | 59400 | 3210 | 24000 |
| IgG (g/L) | 18.73 | 57.23 | 15.51 | 24.59 |
| IgE (KU/L) | 521 | 7.3 | 427 | 5000 |
| Eosinophils (10^9^/L) | 0.3 | 1.08 | 0.12 | 0.37 |
| C3 (g/L) | 0.827 | 0.521 | 1.284 | 0.702 |
| C4 (g/L) | 0.171 | 0.078 | 0.096 | 0.111 |
| ANCA antibodies | Negative | Negative | Negative | Negative |
| **Organ involvements patterns** |  |  |  |  |
|  | Lacrimal gland | Lacrimal gland | Submandibular gland | Lacrimal gland |
|  | Submandibular gland | Parotid gland | Bile duct | Submandibular gland |
|  | Lung | Musculus ocularis | Pancreas | Lung |
|  | Paranasal sinus | Retroperitoneum |  |  |

**Table S3. The detailed clinical features of 9 IgG4-RD patients used for Western blot.**

|  | **Values** | **Normal ranges** |
| --- | --- | --- |
| **Demographic features** | | |
| Sex (Male : Female) | 1.3 : 1 | N. A |
| Age at onset (Mean ± S.D.) | 62.89±11.12 | N. A |
| Disease duration (Month; Median, IQR) | 12 (6-84) | N. A |
| Affected organ numbers (Mean ± S.D.) | 4.11±1.96 | N. A |
| RI scores (Mean ± S.D.) | 8.44±3.97 | N. A |
| Treatment naïve (n%) | 9/9, 100% | N. A |
| **Serological features (Median, IQR)** | | |
| IgG4 (mg/L) | 14800 (8430-18550) | 80-1400 |
| IgG (g/L) | 22.33 (18.85-24.56) | 7.00-17.00 |
| IgA (g/L) | 1.49 (1.37-3.05) | 0.70-4.00 |
| IgM (g/L) | 0.87 (0.81-1.30) | 0.40-2.00 |
| IgE (KU/L) | 286 (186-521) | < 60 |
| Eosinophils (10^9^/L) | 0.2 (0.07-0.29) | 0.02-0.5 |
| ESR (mm/h) | 20 (6-26) | 0-20 |
| CRP (mg/L) | 0.61 (0.26-1.71) | < 8.0 |
| C3 (g/L) | 0.801 (0.649-1.20) | 0.73-1.46 |
| C4 (g/L) | 0.167 (0.067-0.24) | 0.1-0.4 |
| ANCA (Nagative%) | 9/9, 100% | N. A |
| **Organ involvements (n%)** | |  |
| Lacrimal gland | 4 (44.44%) | N. A |
| Parotid gland | 2 (22.22%) | N. A |
| Submandibular gland | 8 (88.89%) | N. A |
| Pancreas | 4 (44.44%) | N. A |
| Bile duct | 1 (11.11%) | N. A |
| Lung | 4 (44.44%) | N. A |
| Kidney | 2 (22.22%) | N. A |
| Retroperitoneum | 1 (11.11%) | N. A |
| Paranasal sinus | 4 (44.44%) | N. A |
| Lymph node | 6 (66.67%) | N. A |

**S.D. represented for Standard deviation, IQR represented for interquartile range.**

**RI scores was the IgG4-RD responder index scores; IgG, IgA, IgM, IgG4, IgE were immunoglobulin G, A, M, G4 and E. ESR was Erythrocyte Sedimentation Rate, CRP was C-reactive protein, RF was rheumatoid factor and C3/4 represented for complement 3/4.**

**Table S4. The detailed clinical features of the 7 IgG4-RD patients used for ELISA.**

|  | **Values** | **Normal ranges** |
| --- | --- | --- |
| **Demographic features** | | |
| Sex (Male : Female) | 2.5 : 1 | N. A |
| Age at onset (Mean ± S.D.) | 55.86±14.16 | N. A |
| Disease duration (Month; Median, IQR) | 24 (1-72) | N. A |
| Affected organ numbers (Mean ± S.D.) | 2.43±1.40 | N. A |
| RI scores (Mean ± S.D.) | 5.43±2.23 | N. A |
| Treatment naïve (n%) | 7/7, 100% | N. A |
| **Serological features (Median, IQR)** | | |
| IgG4 (mg/L) | 8280 (2390-9670) | 80-1400 |
| IgG (g/L) | 16.13 (13.76-32.85) | 7.00-17.00 |
| IgA (g/L) | 2.24 (1.55-3.31) | 0.70-4.00 |
| IgM (g/L) | 0.59 (0.40-1.32) | 0.40-2.00 |
| IgE (KU/L) | 309 (170-1625.5) | < 60 |
| Eosinophils (10^9^/L) | 0.15 (0.115-0.335) | 0.02-0.5 |
| ESR (mm/h) | 16 (8-34) | 0-20 |
| CRP (mg/L) | 2.24 (0.8-2.93) | < 8.0 |
| C3 (g/L) | 0.913 (0.714-1.138) | 0.73-1.46 |
| C4 (g/L) | 0.125 (0.062-0.203) | 0.1-0.4 |
| ANCA (Nagative%) | 7/7, 100% | N. A |
| **Organ involvements (n%)** | |  |
| Lacrimal gland | 4 (57.14%) | N. A |
| Parotid gland | 0 | N. A |
| Submandibular gland | 3 (42.86%) | N. A |
| Pancreas | 1 (14.29%) | N. A |
| Bile duct | 0 | N. A |
| Lung | 0 | N. A |
| Kidney | 0 | N. A |
| Retroperitoneum | 2 (28.57%) | N. A |
| Paranasal sinus | 0 | N. A |
| Lymph node | 5 (71.43%) | N. A |

**S.D. represented for Standard deviation, IQR represented for interquartile range.**

**RI scores was the IgG4-RD responder index scores; IgG, IgA, IgM, IgG4, IgE were immunoglobulin G, A, M, G4 and E. ESR was Erythrocyte Sedimentation Rate, CRP was C-reactive protein, RF was rheumatoid factor and C3/4 represented for complement 3/4.**


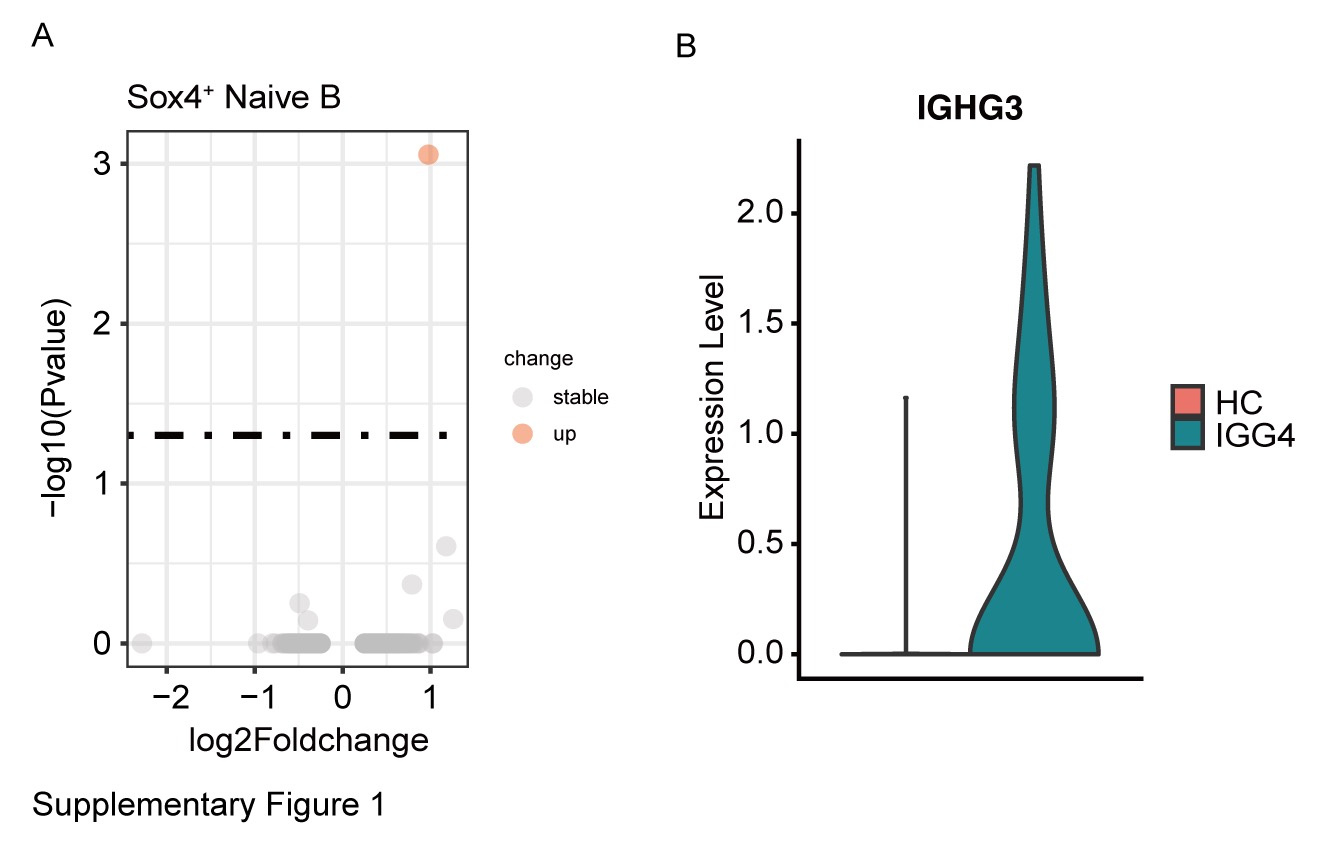


**Supplementary Figure 1 DEGs of Sox4^+^ Naïve B cells.**

1. Volcano plot showing the DEGs of Sox4^+^ Naïve B cells between HCs and IgG4-RD patients.
2. Violin plot showing the *IGHG3* expression in Sox4^+^ Naïve B cells between HCs and IgG4-RD patients.


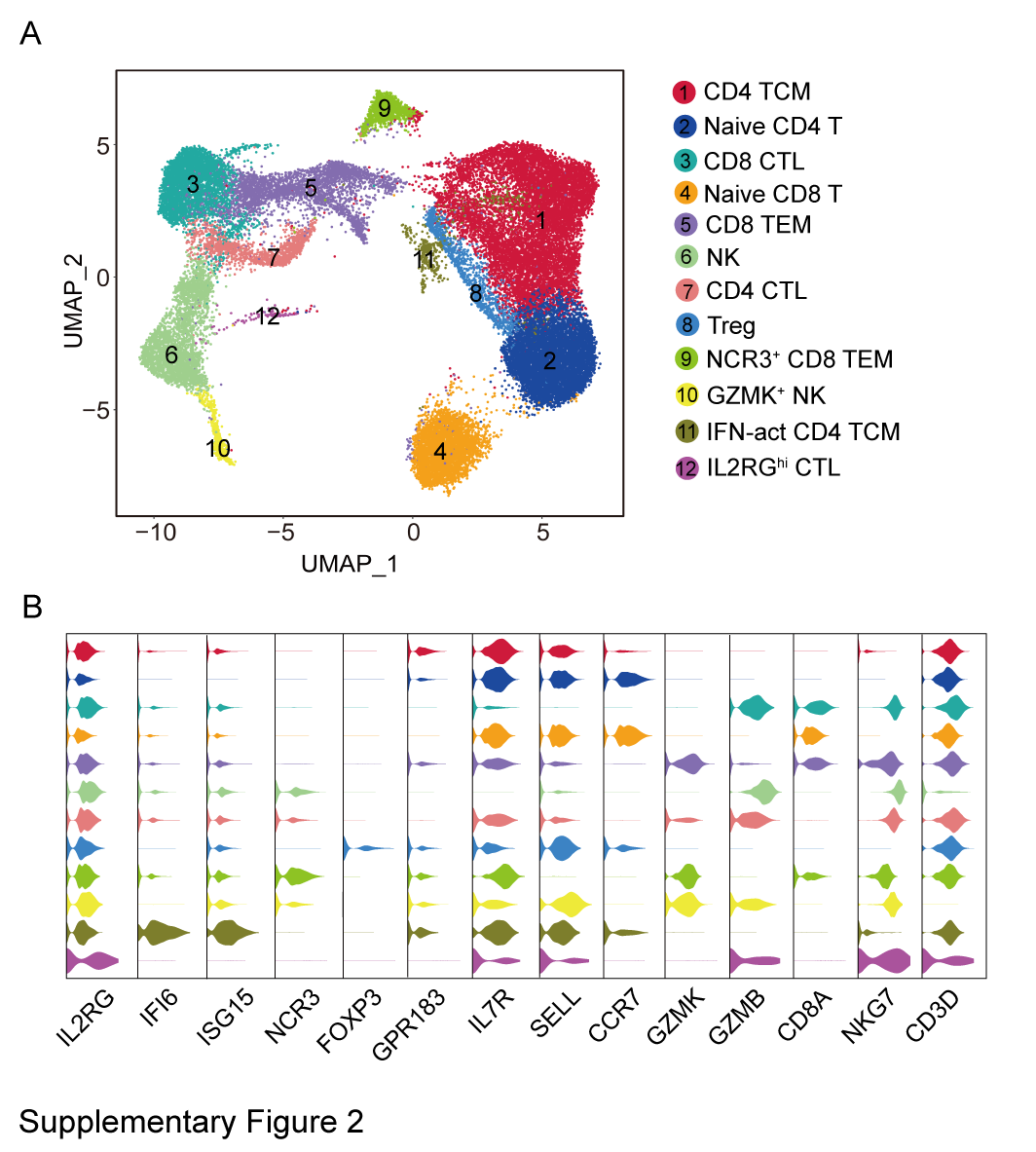


**Supplementary Figure 2 Overview of the clustering and annotation of T/NK subsets.**

1. UMAP representation of 35,309 T/NK cells from HCs (n=3) and IgG4-RD (n=4), showing the formation of 12 clusters.
2. Canonical cell markers are used to label major cell types represented in the violin plot. The legend is labeled in log scale.


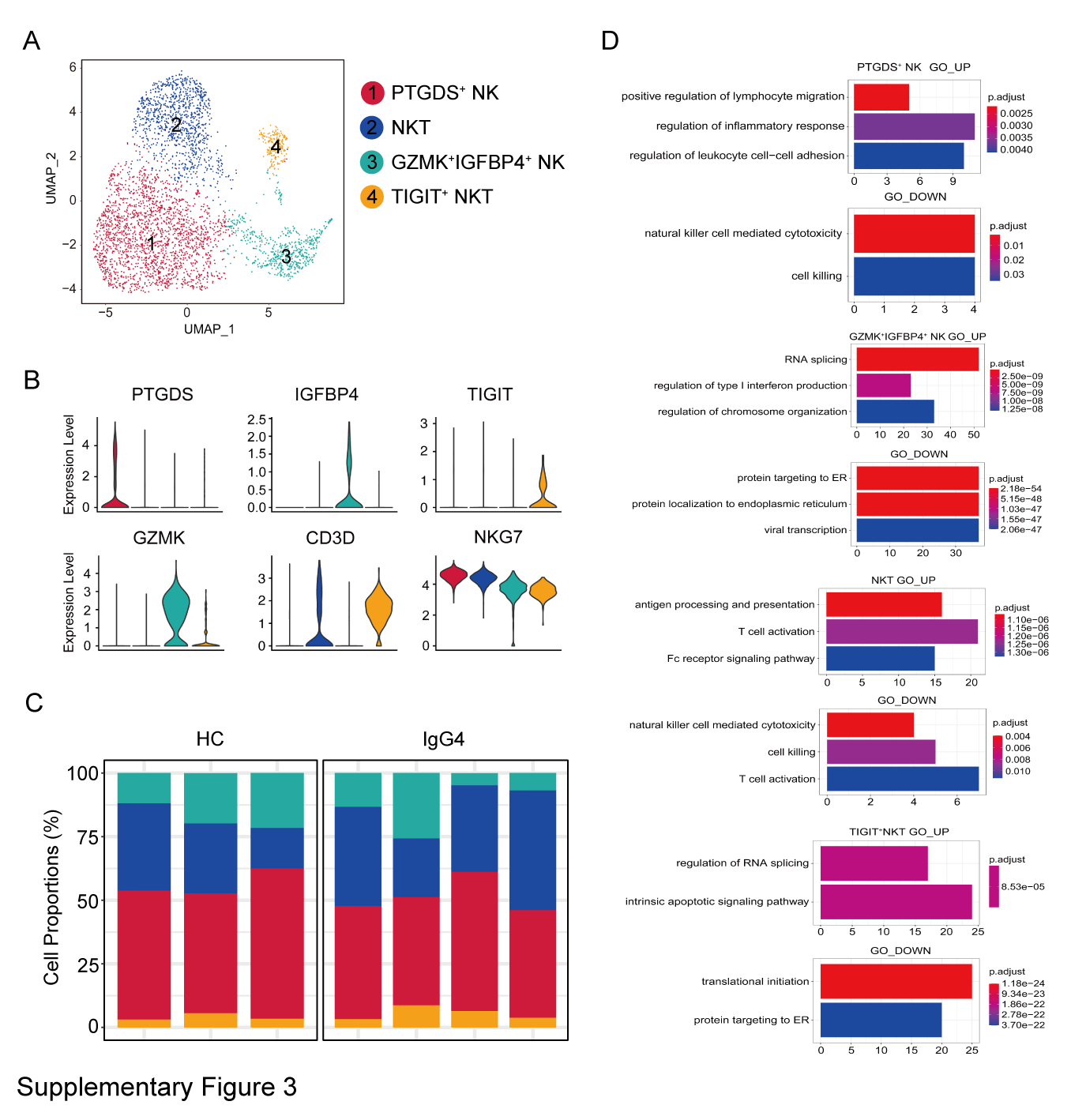


**Supplementary Figure 3 The heterogeneity and transcriptional features of NK cell subsets in IgG4-RD.**

1. UMAP representation of 3699 NK cells, showing the formation of 4 clusters.
2. Violin plots showing expression distribution of canonical cell markers.
3. Bar plot showing cluster abundance of each NK cell types across all samples.
4. GO analysis showing the biological process enriched in NK cell subtypes of IgG4-RD patients.
